# Protective Effects of Quercetin on Anxiety-Like Symptoms and Neuroinflammation Induced by Lipopolysaccharide in Rats

**DOI:** 10.1155/2020/4892415

**Published:** 2020-04-28

**Authors:** Bombi Lee, Mijung Yeom, Insop Shim, Hyejung Lee, Dae-Hyun Hahm

**Affiliations:** ^1^Acupuncture and Meridian Science Research Center, College of Korean Medicine, Kyung Hee University, 02447 Seoul, Republic of Korea; ^2^Department of Physiology, College of Medicine, Kyung Hee University, 02447 Seoul, Republic of Korea

## Abstract

Recently, neuroinflammation is thought to be one of the important causes of many neuropsychiatric diseases. Quercetin (QUER) is a natural flavonoid, and it is well known that QUER has antioxidative, anti-inflammatory, and neuroprotective effects. In our study, lipopolysaccharide (LPS) was injected into the lateral ventricle of rats to induce anxiety-like behaviors and neuroinflammation, and it was confirmed that chronic administration of QUER could improve anxiety-like symptoms. We also investigated the effects of QUER on inflammatory markers and its major mechanisms associated with inflammation in the hippocampus. Daily administration of QUER (10, 50, and 100 mg/kg) daily for 21 days significantly improved anxiety-like behaviors in the elevated plus-maze test and open field test. QUER administration significantly reduced inflammatory markers such as interleukin-6, interleukin-1*β*, cyclooxygenase-2, and nuclear factor-kappaB levels in the brain. In addition, QUER significantly increased the brain-derived neurotrophic factor (BDNF) mRNA level and decreased the nitric oxide synthase (iNOS) mRNA level. Therefore, our results have shown that QUER can improve anxiety-like behaviors caused by chronic neuroinflammation. This anxiolytic effect of QUER has been shown to be due to its anti-inflammatory effects and appropriate regulation of BDNF and iNOS expression. Thus, QUER provides the potential as a therapeutic agent to inhibit anxiety-like symptoms in neuropsychiatric diseases, such as anxiety.

## 1. Introduction

Neuroinflammation is thought to be responsible for multiple psychiatric disorders, such as anxiety, depression, schizophrenia, Alzheimer's disease, and epilepsy in the central nervous system (CNS) [1]. The immune system is easily infected as age progresses [2, 3]. In particular, long-lasting, serious postinfectious sepsis can lead to depression or anxiety in elderly patients [4]. According to various epidemiological studies, neuroinflammation is a major risk factor, and the relation between systemic inflammation and the cause of neuropsychiatric disorders has been reported [1]. Recently, targeting neuroinflammation has been proposed as a novelty therapeutic tool for the control of neuropsychiatric disease [5].

It has been found that it is important to regulate various inflammatory cytokines, such as interleukin-6 (IL-6), interleukin-1*β* (IL-1*β*), and tumor necrosis factor-*α* (TNF-*α*) in neuropsychiatric disease [6]. These cytokines are known to induce behavioral deficits characterized by helplessness, decreased exercise capacity, asthenia, sleep disturbances, and memory and learning disabilities [7].

Experimental exposure of rodents to viral mimetics or bacterial active ingredients, such as lipopolysaccharide (LPS), can enhance anxiety- and depression-like behaviors possibly through the reprogramming of the hypothalamic-pituitary-adrenal axis [8]. In addition, neuroinflammation caused by a single intraperitoneal injection of LPS is known to significantly increase the levels of neuroinflammatory factors, such as cytokines [9]. LPS injection stimulates inflammatory cytokine through the Toll-like receptor 4 (TLR4), which leads to the activation of many inflammatory factors, such as IL-6, IL-1*β*, and the nuclear factor-kappaB (NF-*κ*B) system [10].

Nonsteroidal anti-inflammatory drugs (NSAIDs) are the most extensively used medicines to treat neuroinflammation [11]. These NSAIDs are prescribed to patients because they control inflammation and analgesia [12]. However, sustained use of NSAIDs can lead to gastrointestinal side effects as well as liver and kidney toxicity, which limits its use [13]. These adverse effects triggered a desire for the development of safer novelty therapies for long-term treatment [14]. In a recent study, natural products have been discussed as a new alternative for the treatment of neuroinflammation and anxiety-like symptoms [15].

There has been considerable interest in flavonoids as anxiolytic or antioxidant agents with pharmacological effects [16]. Quercetin (3,3′,4′,5,6-pentahydroxyflavone, QUER) is a polyphenolic system commonly found in apples, strawberries, soybeans, broccoli, grapes, citrus fruits, and tea [12]. QUER has been reported to have many beneficial effects on the diseases, such as antiallergic, antirheumatic, anti-inflammatory, and antiviral effects [17]. QUER inhibits ROS-producing enzymes and is known to prevent neuronal damage induced by oxidative stress [18, 19]. Also, it has been found to exert anxiolytic properties and can improve cognitive functions in neurobehavioral disorders [20]. Some studies have shown that QUER can improve the neurobehavioral and immunological deficits that manifest in adriamycin-treated rats [21]. Also, it has been noted that QUER can easily pass through the blood-brain barrier [19].

The researchers have led to the hypothesis that QUER may also be effective for mitigating LPS-induced neuropsychiatric symptoms, including anxiety. The study investigated the anti-inflammatory effects of QUER. The elevated plus-maze (EPM) test and the open field test (OFT) were performed in order to assess its effects on anxiety-like behaviors impeded by LPS-induced chronic inflammation in rats. Furthermore, we also studied the mechanisms of the QUER effect in neural damage and molecular neurobiological control of neuronal inflammation.

LPS-stimulated inflammatory cytokine cascades have often been used to study the neurobiological mechanisms of anxiety-like behaviors caused by inflammatory mediators and to develop targeted therapies for neuroinflammation in animal models.

## 2. Methods

### 2.1. Animals

Six-week-old, male SD rats (weighting 210–230 g, Samtako Animal Co., Seoul, Korea) were used in this study. The vivarium room was kept on a 12 h light/dark cycle (lights on at 9:00 and lights off at 21:00) under a relative humidity of 50 ± 10% and a controlled temperature of 21 ± 3°C. All rats were caged for 7 days to acclimatize before beginning the experimental protocol. All animal experiments followed the “Guide for the Animal Experiments” edited by the Korean Academy of Medical Sciences and were approved by the Institutional Animal Ethical Committee, Kyung Hee University (KHUASP (SE)-19-050).

### 2.2. Lesion Generation and Lipopolysaccharide Administration

**Figure 1:**
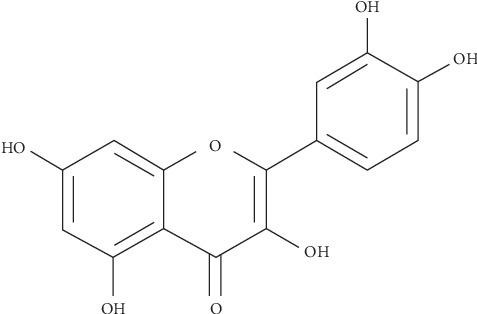
Chemical structure of quercetin.

Quercetin, ibuprofen (IBU), and LPS (*Escherichia coli*; O127 : B8) were purchased from Sigma-Aldrich Company (Sigma-Aldrich Co., St. Louis, MO, USA). The chemical structure of QUER is shown in Figure 1. LPS was administered intracerebroventricularly (i.c.v.) into the lateral ventricle of the rat brain as described previously [3]. Injection of LPS was delivered at a rate of 2 *μ*L/1 min (total 5 min).

**Figure 2:**
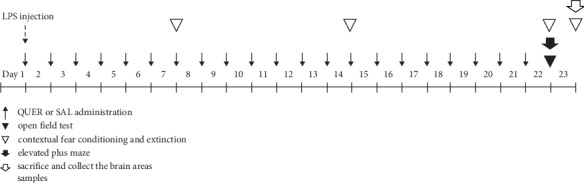
Experimental schedule of lesion generation, quercetin administration, and behavioral tests in rats. LPS: lipopolysaccharide.

The standard dose of LPS in rats and the long-term treatment schedule used in the present study were based on a previous study [3]. QUER and the IBU were administrated intraperitoneally in a volume of 1 ml/kg for 21 days after LPS injection. QUER and IBU were dissolved in 0.9% saline before use. The entire experimental schedule of LPS injection and behavioral examinations is shown in Figure 2.

### 2.3. Elevated Plus-Maze Test

The apparatus consisted of two open arms (50 cm × 10 cm) and two closed arms with dark walls (50 cm × 10 cm × 50 cm). The maze was 50 cm above the ground, and the arms were connected by a platform (10 × 10 cm). Time spent in and numbers of entries into open/closed arms were obtained as anxiolytic-like effects of indices by an observer who was blind to treatment conditions. The protocol was based on a previous study [3].

### 2.4. Open Field Test

In a dimly lit room, each rat was individually placed in a black-painted square wooden box (60 × 60 × 30 cm) and tracked with a video tracking system for 5 minutes. The movement of the rats was measured by the distance (locomotor activity) of the movements observed by the computerized video-tracking analysis program S-MART (PanLab Co., Barcelona, Spain). The number of grooming behaviors (exploration) in the black-painted square wooden box was also manually scored for 5 minutes. The protocol was based on a previous study [3].

### 2.5. Contextual Fear Conditioning and Extinction

Rats were subjected to contextual fear conditioning and extinction following LPS injection. The contextual fear conditioning tests were performed as previously described [3]. Animals were exposed to situational reminders. To stimulate its fear memory, the rats were put in the same container and a tone was activated without shock. Rats were exposed to the tone for 5 minutes on days 7, 14, and 22 because it was previously shown to induce a reexperiencing of the aversive incident and to promote behavioral sensitization. The rate of freezing is divided by total time [12].

### 2.6. IL-6, IL-1*β*, TNF-*α*, COX-2, and NF-*κ*B Measurement

Twenty-three days after LPS injection, IL-6, IL-1*β*, TNF-*α*, cyclooxygenase-2 (COX-2), and NF-*κ*B levels were measured in the hippocampus as based on a previous study [22]. Three rats from each group were severely anesthetized with 1.2% isoflurane and were sacrificed. The hippocampal region was dissected from each of the rats. The levels of IL-6, IL-1*β*, TNF-*α*, COX-2, and NF-*κ*B were assessed through enzyme-linked immunoassay (ELISA) according to the manufacturer's method, and these kits were obtained from Abcam (Cambridge, MA, USA).

### 2.7. Total RNA Isolation and Reverse Transcription-Polymerase Chain Reaction

The expressions of inducible brain-derived neurotrophic factor (BDNF), TLR4, and nitric oxide synthase (iNOS) mRNA using RT-PCR were analyzed according to a previous study [22]. Total RNA was extracted from the hippocampus of each rat using TRIzol reagent. cDNA was then synthesized from 2 *μ*g total RNA on a thermal cycler (MJ Research, Watertown, MA, USA). PCR products were electrophoresed on 1.2% agarose gels and visualized under UV after GelRed (Biotium, Hayward, CA, USA) staining. Quantification of band intensities was performed using Image Master Total Lab (Amersham Pharmacia Biotech, Piscataway, NJ, USA). Data were normalized against glyceraldehyde-3-phosphate dehydrogenase (GAPDH) expression in the corresponding sample.

### 2.8. Statistical Analysis

All results are expressed as mean ± SEM. The data were analyzed with SPSS 13.0 (Chicago, IL, USA) and were analyzed using multiple ways of analysis of variance (ANOVA) and Tukey's *post hoc* tests. Statistical significance at *p* value < 0.05 has been given in symbols in each figure.

## 3. Results

### 3.1. Effects of Quercetin on Body Weight Loss in Lipopolysaccharide-Induced Rats

**Figure 3:**
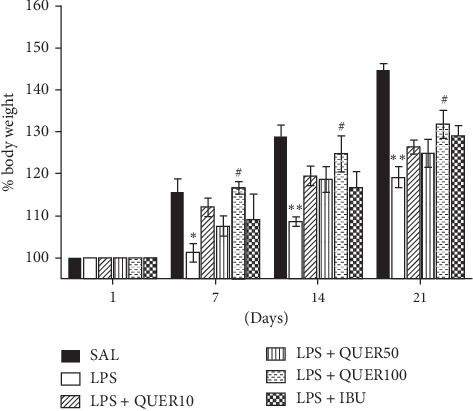
Effects of quercetin on body weight gain on the first day before lipopolysaccharide injection and on days 1, 7, 14, and 21 after lipopolysaccharide injection. ^*∗*^*p* < 0.05, ^*∗∗*^*p* < 0.01 vs. SAL group; ^#^*p* < 0.05 vs. LPS group.

We measured the body weight of each rat in each group on days 1, 7, 14, and 21 (Figure 3). Normal rats gradually had an increase in body weight over time. On the other hand, LPS-lesioned rats began to lose body weight on day 7. On day 1, there was no difference in the body weight of rats in all groups. However, over the course of 7 days, the rats treated with LPS showed a significant decrease in body weight after day 7 (*p* < 0.05) compared with the saline-treated (SAL) group. Also, the rats treated with LPS showed a significant decrease in body weight through days 14 and 21 (*p* < 0.01) compared with the saline-treated (SAL) group. The body weight in the LPS + QUER100 group significantly slowed down body weight loss compared to the LPS group on days 14 and 21 (*p* < 0.05).

### 3.2. Effects of Quercetin on Anxiety-Like Behaviors in Lipopolysaccharide-Induced Rats

**Figure 4:**
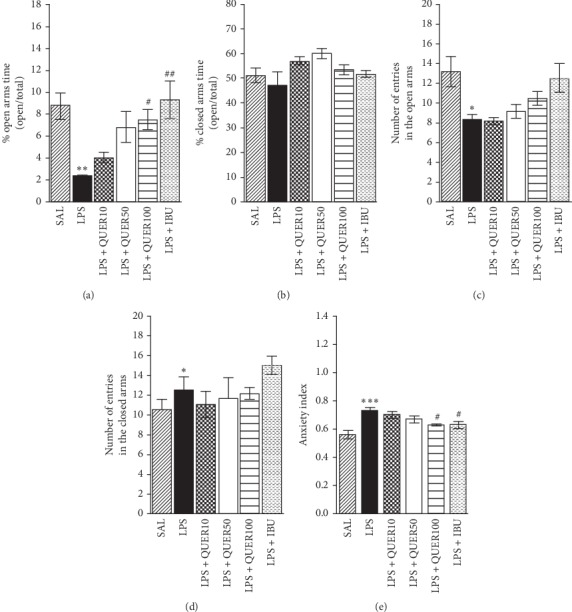
Effects of quercetin administration on the percentage of time spent in the open and closed arms, numbers of entries in the open and closed arms, and anxiety index in the elevated plus-maze test after lipopolysaccharide injection. ^*∗*^*p* < 0.05, ^*∗∗*^*p* < 0.01, ^*∗∗∗*^*p* < 0.001 vs. SAL group; ^#^*p* < 0.05, ^##^*p* < 0.01 vs. LPS group.

Following exposure to LPS, the percentage of time (*F* (5, 35) = 5.890, *p* < 0.01) and entries (*F* (5, 35) = 4.862, *p* < 0.05) into open arms were reduced significantly (Figure 4). Conversely, the percentage of time (*F* (5, 35) = 2.646, *p*=0.55) and entries (*F* (5, 35) = 1.477, *p*=0.227) into closed arms were elevated markedly. We observed that LPS-lesioned animals spent less time in the open arms of the EMP compared with the SAL group (*p* < 0.01). No significant differences in the other parameters (closed arms) were found between groups. However, rats in the LPS + QUER100 group showed significant restoration of the time spent in the open arms of the maze compared with the LPS group (*p* < 0.05). We also observed that LPS-lesioned animals significantly decreased the number of entries in the open arms of the EMP compared with the SAL group (*p* < 0.05). However, rats in the LPS + QUER100 group showed restoration of the number of entries in the open arms of the maze compared with the LPS group, but it was not statistically significant. Overall, the anxiety index is calculated by application of the number of visits to and time spent in the open and closed arms. Therefore, rats in the LPS + QUER100 group showed significantly decreased anxiety index compared with the LPS group (*p* < 0.05).

### 3.3. Effects of Quercetin on Locomotion and Grooming Behavior in Lipopolysaccharide-Induced Rats

**Figure 5:**
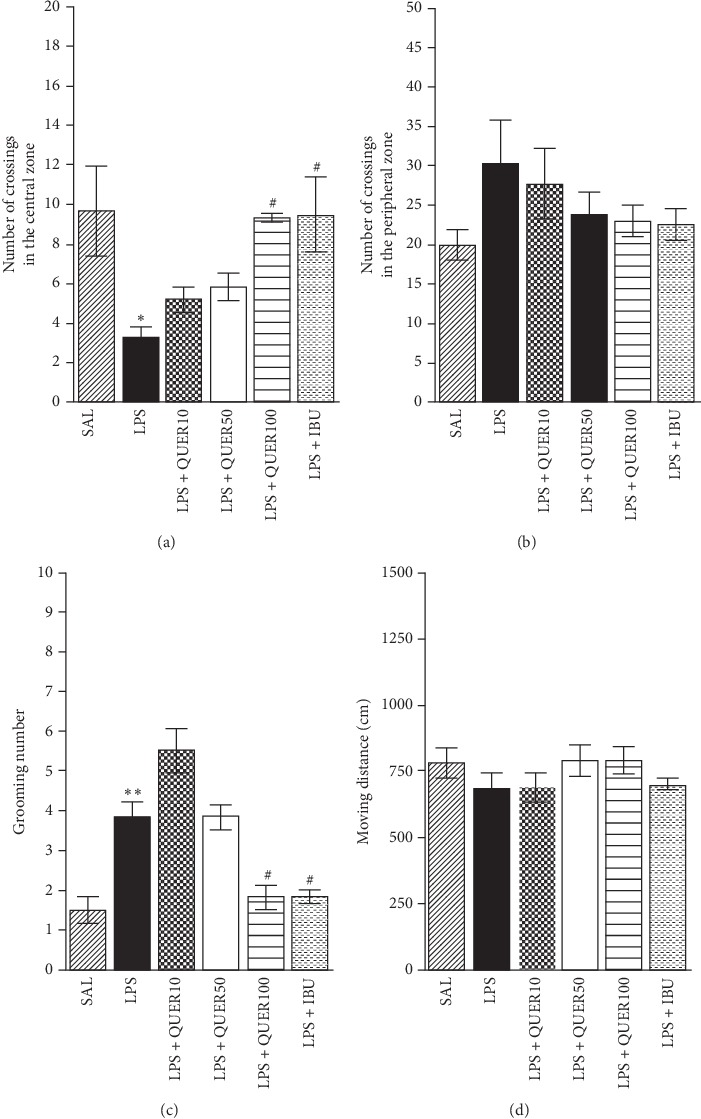
Effects of quercetin administration on locomotion and exploratory behavior in the open field test in rats exposed to lipopolysaccharide. Number of crossings in the central and peripheral zones, locomotor activity, and number of grooming behaviors in the open field test after lipopolysaccharide injection. ^*∗*^*p* < 0.05, ^*∗∗*^*p* < 0.01 vs. SAL group; ^#^*p* < 0.05 vs. LPS group.

Following exposure to LPS, crossing in the central zone was reduced significantly (*p* < 0.05; Figure 5). As shown in Figure 5(b), no significant difference in the number of crossings in the peripheral zone could be observed (*p*=0.319). However, rats treated with 100 mg/kg of QUER showed significant increases in the number of central zone crossings compared with the LPS group (*p* < 0.05). Conversely, the duration of grooming was elevated after exposure to LPS (*F* (5, 35) = 18.630, *p* < 0.05). However, rats in the LPS + QUER100 group showed significantly decreased grooming behavior compared with the LPS group in the open field (*p* < 0.05). This study showed that LPS treatment did not affect the distance of locomotion and evidence that QUER treatment also did not affect motor functions and exploration behaviors (Figure 5(d)). This demonstrated that the rats' behavior is caused by anxiety-like symptoms and not by other pathological factors or side effects, such as motor function.

### 3.4. Effects of Quercetin on Contextual Freezing Behavior in Lipopolysaccharide-Induced Rats

**Figure 6:**
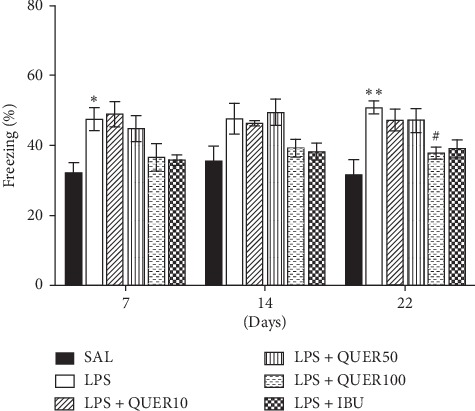
Effects of quercetin on freezing behavior after exposure to lipopolysaccharide in rats. The percentages of time spent freezing were determined on days 7, 14, and 22. ^*∗*^*p* < 0.05, ^*∗∗*^*p* < 0.01 vs. SAL group; ^#^*p* < 0.05 vs. LPS group.

Next, we examined contextual freezing behavior in rats after the injection of LPS. Freezing time was significantly enhanced after injection of LPS (*p* < 0.05 on day 7 and *p* < 0.01 on day 22), respectively (Figure 6). 100 mg/kg of QUER did not reduce the increase in freezing time on days 7 and 14, but significantly decreased the freezing time on day 22 (*p* < 0.05). These results showed that the continuous fear response to the original situation is associated with neuroinflammation of LPS injection, but this fear response is weakened by the QUER treatment.

### 3.5. Effects of Quercetin on Inflammatory Mediators of the Hippocampus in Lipopolysaccharide-Induced Rats

**Figure 7:**
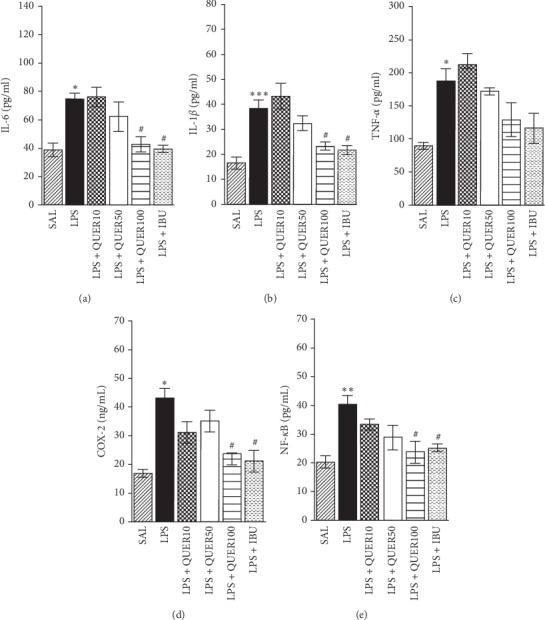
Effects of quercetin on interleukin-6 (IL-6), interleukin-1*β* (IL-1*β*), tumor necrosis factor-*α* (TNF-*α*), cyclooxygenase-2 (COX-2), and nuclear factor-kappaB (NF-*κ*B) concentrations in the brains after lipopolysaccharide injection. ^*∗*^*p* < 0.05, ^*∗∗*^*p* < 0.01, ^*∗∗∗*^*p* < 0.001 vs. SAL group; ^#^*p* < 0.05 vs. LPS group.

Figure 7 shows the investigation of the levels of proinflammatory markers in the hippocampus activated by LPS-induced neuroinflammation. The effect of QUER administration on these markers was also investigated. The hippocampal levels of proinflammatory markers, IL-6, IL-1*β*, TNF-*α*, COX-2, and NF-*κ*B, were compared and analyzed.

*Post hoc* test results revealed significantly higher IL-6 level in the hippocampus of the LPS group than in the SAL group (*p* < 0.05). Daily administration of 100 mg/kg of QUER significantly attenuated the LPS-induced increase of IL-6 in the hippocampus (*p* < 0.05). Similar to IL-6, *post hoc* test results revealed higher IL-1*β* levels in the hippocampus of the LPS group than in the SAL group (*p* < 0.001). Daily administration of 100 mg/kg of QUER attenuated the LPS-induced increase in IL-1*β* in the hippocampus (*p* < 0.05). Similar to IL-6, *post hoc* test results revealed significantly higher TNF-*α* level in the hippocampus of the LPS group than in the SAL group (*p* < 0.05). Daily administration of 100 mg/kg of QUER attenuated the LPS-induced increase of TNF-*α* in the hippocampus, but it was not statistically significant. Treatment with 100 mg/kg of QUER significantly rescued the COX-2 level in the hippocampus by 54.89%, relative to untreated controls (*p* < 0.05). Treatment with 100 mg/kg of QUER significantly rescued NF-*κ*B level in the hippocampus by 58.74%, relative to untreated controls (*p* < 0.05).

### 3.6. Effects of Quercetin on Expression of iNOS, TLR4, and BDNF mRNA of the Hippocampus in Lipopolysaccharide-Induced Rats

**Figure 8:**
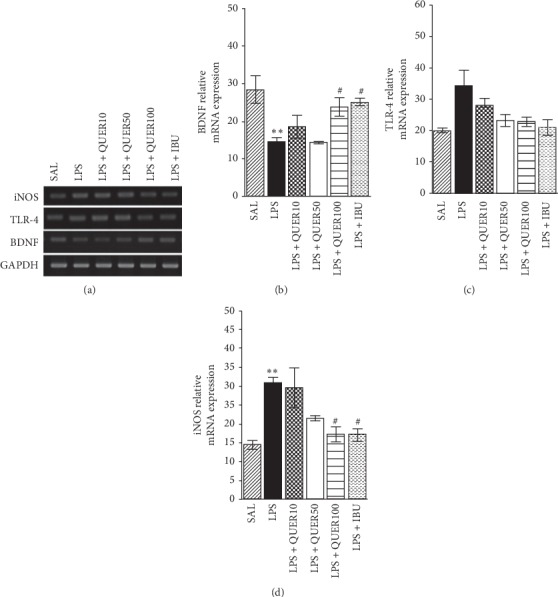
Effects of quercetin on the expression of brain-derived neurotrophic factor (BDNF), Toll-like receptor 4 (TLR4), and inducible nitric oxide synthase (iNOS) mRNAs in rats with lipopolysaccharide-induced hippocampal impairment. The expression levels of BDNF, TLR4, and iNOS mRNAs were normalized relative to glyceraldehyde 3-phosphate dehydrogenase (GAPDH) mRNA as an internal control. ^*∗∗*^*p* < 0.01 vs. SAL group; ^#^*p* < 0.05 vs. LPS group.

We also evaluated the effect of chronic QUER treatment on the BDNF expression in the hippocampus of rats. Chronic treatment of rats with QUER (100 mg/kg) inhibited the decrease of BDNF mRNA expression in the hippocampus (*p* < 0.05; Figure 8).

PCR analysis found that LPS injection resulted in increases in the TLR4 mRNA (172.02%) expression in the hippocampus compared to the levels in rats in the SAL group, but it was not statistically significant. Administration of 100 mg/kg of QUER inhibited these LPS-induced increases in the TLR4 mRNA levels in the hippocampus, but it was not statistically significant.

PCR analysis found that LPS injection resulted in significant increases in the iNOS mRNA (213.98%) expression in the hippocampus compared to the levels in rats in the SAL group (*p* < 0.01). Administration of 100 mg/kg of QUER significantly inhibited these LPS-induced increases in the iNOS mRNA levels in the hippocampus (*p* < 0.05).

## 4. Discussion

The data presented here highlight the strong anxiolytic effects of QUER in a rat model of anxiety-like symptoms and provide evidence of the potential mechanisms underlying these effects. Treatment with QUER improved LPS-induced anxiety-like behaviors in rats. Anxiety-like symptoms can be caused by the administration of a neuroinflammatory substance [14]. Injection of LPS of rats causes anxiety-like behaviors and was the basis for the animal model in this study [14]. LPS injection into the rats was performed followed by continuous QUER treatment. QUER treatment significantly increased the time spent in the open arms in the EPM and reduced the anxiety index of the LPS-induced rats. In addition, administration of QUER after LPS infiltration also significantly increased the number of central zone crossings in the OFT. QUER also significantly inhibited the increase of IL-6 and IL-1*β*, which are neuroinflammatory cytokines, in the hippocampus. In addition, administration of QUER significantly increased the levels of decreased BDNF mRNA levels and reduced the levels of increased iNOS mRNA levels in the hippocampus. Thus, QUER has demonstrated that adequate modulation of BDNF and iNOS can significantly alleviate LPS-induced anxiety-like behaviors. Thus, QUER is an effective anti-inflammatory and a potentially useful neuroprotective agent because it significantly inhibits the production of inflammatory cytokines. The administration of QUER produced anxiolytic-like effects in an animal model of anxiety. We also investigated the dose-dependent effects of QUER. Optimal efficacy was obtained at a dose of at least 100 mg/kg, and this dose was most effective in this study as well as in previous studies [23].

In the present study, administration of QUER after LPS injection significantly restored reduced body weight, indicating that QUER can inhibit physiological changes and anxiety-like symptoms due to LPS-induced chronic inflammation [2].

Furthermore, the results of our behavioral investigation have demonstrated the anxiolytic-like effects of QUER in an animal model of anxiety. In the EPM test, the administration of QUER after LPS injection significantly reduced anxiety-like behaviors, as indicated by exploratory behaviors, indicating that rats receiving QUER had more entries in the open arms than those receiving LPS. In the EPM test, QUER has an anxiolytic effect on LPS-related psychological symptoms, such as anxiety, and thus, our results indicate that QUER may inhibit anxiety-like manifestations due to neuroinflammation. Furthermore, QUER administration after injection of LPS significantly increased the number of central zone crossings in the OFT.

There was no significant individual difference in the locomotor activity between groups in the results of the behavioral activity of anxiety in the OFT. Thus, it has been shown that the administration of QUER had no effect on sensorimotor performance. However, the administration of QUER after injection of LPS significantly increases the number of central zone crossings in the OFT, thereby significantly reducing anxiety-like behaviors. Administration of QUER may be due to anxiolytic action. In this study, as in previous studies, injection of LPS appears to that freezing time is enhanced by impairing contextual fear conditioning of the context. However, we have confirmed that QUER could alleviate the fear memory and anxiety caused by the injection of LPS. Therefore, in this study, a variety of behavioral tests have demonstrated that rats after injection of LPS have shown behavioral changes, contextual fear conditioning, and anxiety-like behavior. Therefore, QUER treatment has shown that it could alleviate the anxiety-like behavior in LPS-treated rats, which has been shown through the reduction of freezing time in the contextual fear conditioning and extinction.

Our study has shown that LPS administration significantly increased the IL-6, IL-1*β*, and TNF-*α* expression levels in the hippocampus. The expression of TNF-*α* and IL-1*β* mRNAs is dynamically regulated by various immune cells in the inflammatory response of the hippocampus [24]. However, QUER has shown the potential to improve abnormalities, such as anxiety-like behaviors, and neuroinflammation by continuously reducing the levels of IL-6 and IL-1*β* induced by LPS. In addition, these results also showed that the inflammatory reactions to LPS infiltration significantly increased COX-2 level by regulating the NF-*κ*B pathway in the hippocampus. Previous studies have also shown that NF-*κ*B, which primarily regulates the inflammatory reactions, is activated by infiltration of LPS [25]. In the present study, QUER restored LPS-induced behavior disturbances, such as anxiety by significantly inhibiting COX-2 levels through the modulation of NF-*κ*B.

We also analyzed the activation of the LPS receptor TLR4 and iNOS, which are involved in the production of proinflammatory cytokines and activation of NF-*κ*B and inflammatory mediators. Long-term treatment of LPS has been shown to activate the NF-*κ*B other inflammatory pathways through TLR4 and increase the expression of inflammatory cytokines in the hippocampus [26]. Our results have shown that QUER could alleviate the induction of proinflammatory cytokines, such as IL-6 and IL-1*β*, and iNOS expression by LPS via these pathways. Thus, inhibition of iNOS activation by the administration of QUER during chronic inflammation may suggest the possibility of preventing neuropsychiatry disease [27].

We also examined the BDNF mRNA levels in the hippocampus. Since BDNF is involved in the induction of anxiety-like symptoms in the hippocampus, regulation of BDNF signaling may be an important factor in the anxiety-like behavior of rats induced by LPS injection [28]. In the present study, QUER treatment significantly reversed the reduction of BDNF mRNA expression by LPS injection, demonstrating that the beneficial effects of QUER can treat anxiety-like behavior by increasing BDNF mRNA expression.

## 5. Conclusion

As a result, QUER administration significantly reduced neuroinflammation through the modulation of BDNF and iNOS, thereby improving the anxiety-like symptoms stimulated by LPS injection on the behavioral tests. These findings suggest that QUER may improve the psychologically rooted behaviors and neurochemical alterations seen in anxiety-like symptoms. Based on these data, QUER presents the potential as a useful alternative therapy for the treatment of neuropsychiatric disorders, such as anxiety.

## Data Availability

The original data used to support the findings of this study are included within the article.

## References

[1] Radtke F. A., Chapman G., Hall J., Syed Y. A. (2017). Modulating neuroinflammation to treat neuropsychiatric disorders. *BioMed Research International*.

[2] Jiang X., Wang G., Lin Q., Tang Z., Yan Q., Yu X. (2019). Fucoxanthin prevents lipopolysaccharide-induced depressive-like behavior in mice via AMPK-NF-*κ*B pathway. *Metabolic Brain Disease*.

[3] Lee B., Shim I., Lee H., Hahm D. H. (2018). Gypenosides attenuate lipopolysaccharide-induced neuroinflammation and anxiety-like behaviors in rats. *Animal Cells and Systems*.

[4] Holmgren S., Hjorth E., Schultzberg M. (2014). Neuropsychiatric symptoms in dementia-a role for neuroinflammation?. *Brain Research Bulletin*.

[5] Cunningham C., Hennessy E. (2015). Co-morbidity and systemic inflammation as drivers of cognitive decline: new experimental models adopting a broader paradigm in dementia research. *Alzheimer’s Research & Therapy*.

[6] Todorović N., Filipović D. (2017). The antidepressant- and anxiolytic-like effects of fluoxetine and clozapine in chronically isolated rats involve inhibition of hippocampal TNF-*α*. *Pharmacology Biochemistry and Behavior*.

[7] Mrak R. E. (2009). Neuropathology and the neuroinflammation idea. *Journal of Alzheimer’s Disease*.

[8] Ellis S., Mouihate A., Pittman Q. J. (2005). Early life immune challenge alters innate immune responses to lipopolysaccharide: implications for host defense as adults. *The FASEB Journal*.

[9] Andy S. N., Pandy V., Alias Z., Kadir H. A. (2018). Deoxyelephantopin ameliorates lipopolysaccharides (LPS)-induced memory impairments in rats: evidence for its anti-neuroinflammatory properties. *Life Sciences*.

[10] Mirahmadi S. M., Shahmohammadi A., Rousta A. M. (2017). Soy isoflavone genistein attenuates lipopolysaccharide-induced cognitive impairments in the rat via exerting anti-oxidative and anti-inflammatory effects. *Cytokine*.

[11] Szekely C. A., Breitner J. C., Fitzpatrick A. L. (2008). NSAID use and dementia risk in the cardiovascular health study: role of APOE and NSAID type. *Neurology*.

[12] Shinozaki T., Yamada T., Nonaka T., Yamamoto T. (2015). Acetaminophen and non-steroidal anti-inflammatory drugs interact with morphine and tramadol analgesia for the treatment of neuropathic pain in rats. *Journal of Anesthesia*.

[13] Graupera M., García-Pagán J. C., Abraldes J. G. (2003). Cyclooxygenase-derived products modulate the increased intrahepatic resistance of cirrhotic rat livers. *Hepatology*.

[14] Yang T. Y., Jang E. Y., Ryu Y. (2017). Effect of acupuncture on Lipopolysaccharide-induced anxiety-like behavioral changes: involvement of serotonin system in dorsal Raphe nucleus. *BMC Complementary and Alternative Medicine*.

[15] Mokhtarian A., Esfandiari E., Ghanadian M., Rashidi B., Vatankhah A. M. (2018). The effects of *Acorus calamus L*. in preventing memory loss, anxiety, and oxidative stress on lipopolysaccharide-induced neuroinflammation rat models. *International Journal of Preventive Medicine*.

[16] Pu F., Mishima K., Irie K. (2007). Neuroprotective effects of quercetin and rutin on spatial memory impairment in an 8-arm radial maze task and neuronal death induced by repeated cerebral ischemia in rats. *Journal of Pharmacological Sciences*.

[17] Bhutada P., Mundhada Y., Bansod K. (2010). Ameliorative effect of quercetin on memory dysfunction in streptozotocin-induced diabetic rats. *Neurobiology of Learning and Memory*.

[18] Ansari M. A., Abdul H. M., Joshi G., Opii W. O., Butterfield D. A. (2009). Protective effect of quercetin in primary neurons against Abeta(1-42): relevance to Alzheimer’s disease. *The Journal of Nutritional Biochemistry*.

[19] Heo H. J., Lee C. Y. (2004). Protective effects of quercetin and vitamin C against oxidative stress-induced neurodegeneration. *Journal of Agricultural and Food Chemistry*.

[20] Aguirre-Hernández E., González-Trujano M. E., Martínez A. L. (2010). HPLC/MS analysis and anxiolytic-like effect of quercetin and kaempferol flavonoids from *Tilia americana* var. *mexicana*. *Journal of Ethnopharmacology*.

[21] Merzoug S., Toumi M. L., Tahraoui A. (2014). Quercetin mitigates Adriamycin-induced anxiety- and depression-like behaviors, immune dysfunction, and brain oxidative stress in rats. *Naunyn-Schmiedeberg’s Archives of Pharmacology*.

[22] Lee B., Yeom M., Shim I., Lee H., Hahm D. H. (2020). Inhibitory effect of carvacrol on lipopolysaccharide-induced memory impairment in rats. *The Korean Journal of Physiology & Pharmacology*.

[23] Tang J., Lu L., Liu Y. (2019). Quercetin improve ischemia/reperfusion-induced cardiomyocyte apoptosis in vitro and in vivo study via SIRT1/PGC-1*α* signaling. *Journal of Cellular Biochemistry*.

[24] Jin Y., Peng J., Wang X., Zhang D., Wang T. (2017). Ameliorative effect of ginsenoside Rg1 on lipopolysaccharide-induced cognitive impairment: role of cholinergic system. *Neurochemical Research*.

[25] Gong Q. H., Pan L. L., Liu X. H., Wang Q., Huang H., Zhu Y. Z. (2011). S-propargyl-cysteine (ZYZ-802), a sulphur-containing amino acid, attenuates beta-amyloid-induced cognitive deficits and pro-inflammatory response: involvement of ERK1/2 and NF-*κ*B pathway in rats. *Amino Acids*.

[26] Palsson-McDermott E. M., O’Neill L. A. (2004). Signal transduction by the lipopolysaccharide receptor, Toll-like receptor-4. *Immunology*.

[27] Kovaiou R. D., Herndler-Brandstetter D., Grubeck-Loebenstein B. (2007). Age-related changes in immunity: implications for vaccination in the elderly. *Expert Reviews in Molecular Medicine*.

[28] Hritcu L., Gorgan L. D. (2014). Intranigral lipopolysaccharide induced anxiety and depression by altered BDNF mRNA expression in rat hippocampus. *Progress in Neuro-Psychopharmacology and Biological Psychiatry*.

